# Perception versus polysomnographic assessment of sleep in CFS and non-fatigued control subjects: results from a population-based study

**DOI:** 10.1186/1471-2377-7-40

**Published:** 2007-12-05

**Authors:** Matthias Majer, James F Jones, Elizabeth R Unger, Laura Solomon Youngblood, Michael J Decker, Brian Gurbaxani, Christine Heim, William C Reeves

**Affiliations:** 1 Department of Psychiatry and Behavioral Sciences, Emory University School of Medicine, Atlanta, USA; 2 Chronic Viral Diseases Branch, Centers for Disease Control & Prevention, Atlanta, USA; 3 Fusion Sleep, Suwanee, USA

## Abstract

**Background:**

Complaints of unrefreshing sleep are a prominent component of chronic fatigue syndrome (CFS); yet, polysomnographic studies have not consistently documented sleep abnormalities in CFS patients. We conducted this study to determine whether alterations in objective sleep characteristics are associated with subjective measures of poor sleep quality in persons with CFS.

**Methods:**

We examined the relationship between perceived sleep quality and polysomnographic measures of nighttime and daytime sleep in 35 people with CFS and 40 non-fatigued control subjects, identified from the general population of Wichita, Kansas and defined by empiric criteria. Perceived sleep quality and daytime sleepiness were assessed using clinical sleep questionnaires. Objective sleep characteristics were assessed by nocturnal polysomnography and daytime multiple sleep latency testing.

**Results:**

Participants with CFS reported unrefreshing sleep and problems sleeping during the preceding month significantly more often than did non-fatigued controls. Participants with CFS also rated their quality of sleep during the overnight sleep study as significantly worse than did control subjects. Control subjects reported significantly longer sleep onset latency than latency to fall asleep as measured by PSG and MSLT. There were no significant differences in sleep pathology or architecture between subjects with CFS and control subjects.

**Conclusion:**

People with CFS reported sleep problems significantly more often than control subjects. Yet, when measured these parameters and sleep architecture did not differ between the two subject groups. A unique finding requiring further study is that control, but not CFS subjects, significantly over reported sleep latency suggesting CFS subjects may have an increased appreciation of sleep behaviour that may contribute to their perception of sleep problems.

## Background

Chronic fatigue syndrome (CFS) is a complex illness defined by unexplained persistent or relapsing fatigue for ≥ 6 months that is not attributable to exertion and is not improved by rest. The fatigue must be accompanied by at least 4 of 8 defining symptoms (significant worsening of fatigue following exertion, unrefreshing sleep, impaired memory or concentration, muscle pain, joint pain, headache, tender cervical or axillary nodes, and sore throat) and the illness must cause substantial functional impairment [1]. Nearly all individuals with CFS report unrefreshing sleep at the time of diagnosis [2-6] and self-reported sleep problems distinguish CFS cases from matched non-fatigued control subjects [7]. In addition, complaints of non-refreshing sleep and difficulty getting to sleep or staying asleep remain common (decreasing from 95.4% to 79.2% and 81.4% to 75%, respectively, when CFS subjects are studied at 3 yearly time points after diagnosis [8]. These complaints and their duration satisfy the definition for chronic insomnia as defined in an NIH Consensus Science Statement [9]. However, while sleep complaints are a prominent component of CFS, major primary sleep disorders (narcolepsy and sleep apnea) are exclusionary medical conditions that preclude the research case definition of CFS [1,10].

Further, polysomnographic studies have not consistently documented sleep abnormalities in people with CFS [11,12]. These observations raise the possibility that people with CFS perceive the quality of their sleep differently from well individuals; i.e., the prominence of self-reported sleep difficulties in CFS may reflect a heightened awareness of altered sleep physiology. Altered self-perception (sensitivity to internal signals) has been suggested to play a role in CFS [13], but few studies have explored the relationship between self-reported sleep quality and objective polysomnographic sleep parameters in persons with CFS. Fossey et al., 2004, contrasted sleep parameters obtained by polysomnography and sleep diaries, medical diagnoses, and results of structured interview and self-report measures between clinic-based subjects with CFS or narcolepsy, and those with no medical or psychiatric diagnoses. Their analyses, which included CFS subjects with sleep disorders identified by PSG and presence of insomnia, described the typical symptom and impairment profiles of the syndrome in CFS patients [14]. A study of twins discordant for CFS found that those with CFS were significantly more likely to report insomnia and daytime sleepiness than their healthy siblings yet night time polysomnographic measurements and multiple sleep latency test (MSLT) did not differ between the groups. This led the authors to speculate that twins with CFS suffered from sleep-state misperception insomnia according to the 1990 International Classification of Sleep Disorders [14-16]. The term sleep-state misperception insomnia has been replaced by the term paradoxical insomnia, which describes paradoxical relationships between objective and subjective sleep assessments in such patients according to the 2005 coding manual [17,18].

In the present study, we evaluated the relationship between subjective and objective measures of sleep alterations in persons with CFS and non-fatigued controls. As detailed previously [12], we conducted overnight polysomnographic studies and daytime multiple sleep latency evaluation of 43 individuals with CFS and 43 non-fatigued controls. The study also included measures of participants' long term and short-term subjective reports of sleep quality. The following questions were addressed: 1) Are subjective sleep problems characteristic of CFS? 2) Is there objective evidence of abnormalities of sleep in CFS as defined by polysomnography? And, 3) Are there associations between subjective sleep problems and objective sleep abnormalities in persons with CFS? To avoid referral bias, a major limitation of studies that recruit CFS subjects from specialty clinics, we enrolled persons with CFS and non-fatigued controls identified from the general population of Wichita, Kansas [19,20]. We also employed standardized criteria to define CFS and controlled for the use of medications known to affect sleep.

## Methods

### Participants

This study adhered to U.S. Department of Health and Human Services human experimentation guidelines and received Institutional Review Board approval from the CDC and collaborating institutions. All participants gave informed consent.

Between January and July 2003, we conducted a 2-day in-hospital study of adults identified with CFS from the general population of Wichita [19]. The in-hospital study enrolled people who participated in the 1997 through 2000 Wichita Population-Based CFS Surveillance Study [20]. The primary objective of the Surveillance Study was to estimate the baseline prevalence and 1-year incidence of CFS in Wichita, Kansas. Participants in the in-hospital study were fatigued adults with medically/psychiatrically unexplained chronic fatigue identified during the surveillance study. Fifty-eight participants had been diagnosed at least once with CFS and 59 had unexplained chronic fatigue that was not CFS. Controls were randomly selected from the cohort who participated throughout surveillance, who did not have medical or psychiatric exclusions, and who had not reported fatigue of at least 1-month duration; they were matched to CFS cases on sex, age, race/ethnicity, and body mass index. Upon admission to this study, subjects were re-evaluated for CFS symptoms and exclusionary medical and psychiatric conditions (discussed below). The 43 who, at the time of the in-hospital study, met 1994 criteria for CFS (discussed below) comprise the cases in this report. Control subjects were 43 individuals who had never reported fatigue during the surveillance study, who were not fatigued at the time of entry into this in-hospital study and who had no exclusionary medical or psychiatric condition identified at the time of the study (following section). Because current classification of CFS was not completely in accord with recruitment classification, strict matching was not maintained, though cases and controls were demographically comparable. Thirty-six (84%) of the 43 with CFS and 38 (88%) of the 43 controls were women; most (40 CFS and 42 controls) were white; their mean ages were 50.6 and 50.3 years, respectively; and body mass index was 29.4 and 29.3, respectively.

### Assessment and classification of CFS

We classified participants as having CFS at the time of the study based on the empirical application [19] of the 1994 CFS research case definition [1]. We used the Multidimensional Fatigue Inventory (MFI) [21] to evaluate fatigue status; we measured functional impairment with the Medical Outcomes Survey short form-36 (SF-36) [22]; and, we used the CDC Symptom Inventory [23] to assess frequency and severity of the 8 CFS defining symptoms. We defined severe fatigue as ≥ medians of the MFI general fatigue (≥ 13) or reduced activity (≥ 10) scales. We defined substantial functional impairment as scores lower than the 25^th ^percentile of published US population on the physical function (≤ 70), or role physical (≤ 50), or social function (≤ 75), or role emotional (≤ 66.7) subscales of the SF-36. Finally, subjects reporting ≥ 4 symptoms and scoring ≥ 25 on the Symptom Inventory Case Definition Subscale were considered to have substantial accompanying symptoms.

To assess whether medical conditions exclusionary for CFS (including untreated hypothyroidism, sleep apnea, or narcolepsy) had developed since the surveillance study, participants provided a standardized past medical history and a listing of current medications, underwent a standardized physical examination, and provided blood and urine for routine analysis. Medications that affect sleep were considered 'sleep medications' for the purpose of analysis and include: *primary hypnotics *(zolpidem, temazepam), *narcotic analgesics *(e.g., hydrocodone, oxycodone, propoxyphene), *anti-depressants *(e.g., citalopram, amitriptyline, imipramine, escitalopram, bupropion, venlafaxine, sertraline, paroxetine, fluoxetine), *anti-anxiety *(alprazolam), *anti-histamines *(e.g., diphenhydramine, chlorpheneramine, promethazine), *decongestants *(e.g., pseudoephedrine, guaifenesin), *anti-convulsants *(e.g., topiramate, clonazepam), *anti-sleep phase disorder *(melatonin), *blood pressure controlling *(e.g., clonidine, midodrine), *anti-psychotics *(e.g., quetiapine, ziprasidone), *stimulants *(e.g., methylphenidate, modafinil), *peristaltic stimulants *(metoclopramide), and *muscle relaxants *(cyclobenzaprine).

To identify psychiatric conditions exclusionary for CFS (current melancholic depression, current and lifetime bipolar disorder or psychosis, substance abuse within 2 years and eating disorders within 5 years), licensed and specifically trained psychiatric interviewers administered the Diagnostic Interview Schedule for Axis I psychiatric disorders.

We classified participants meeting the 3 criteria (MFI, SF-36, and Symptom Inventory) for CFS and in whom no exclusionary medical (including sleep) or psychiatric conditions were identified as having CFS. Participants whose scores were in the normal range on all of the above mentioned instruments and who had no exclusionary medical or psychiatric conditions identified were classified as non-fatigued. Persons with exclusionary medical or psychiatric conditions were not included in the analysis.

### Objective measures of sleep alterations

Sleep studies were conducted in a 4-bed clinical research unit at Wesley Medical Center, Wichita, Kansas [12]. These sleep studies consisted of polysomnography on night #1, Multiple Sleep Latency Tests (MSLT) during the following day, and repeat polysomnography on night #2. Patients were asked to arrive 3 hours before their typical bedtime on night #1 to allow adequate time for electrode application and standard bio-calibrations. "Lights out" and "Lights on" time were 22:00 and 7:00, respectively. MSLT began at 11:00 the following morning and consisted of three additional naps at 13:00, 15:00, and 17:00.

Daytime sleepiness was measured with the MSLT, which has demonstrated objective sensitivity to the effects of sleep deprivation, sleep fragmentation, sleep restriction, insufficient sleep hypersomnia, and in disease states such as sleep apnea and narcolepsy [24,25]. Multiple sleep latency tests were performed and scored according to standard guidelines [26,27] with the exception that four naps were recorded. The mean sleep latency on the MSLT was defined as the mean time from lights out to the first 30-second epoch scored as sleep. A sleep onset REM was defined as one or more epochs of REM sleep occurring within 15 minutes of the first epoch scored as sleep. We considered a mean sleep latency <5 min as pathological sleepiness, scores between 5–10 min as a degree of daytime sleepiness (borderline abnormal), and scores of 10–20 min as normal and a lack of daytime sleepiness. Because mean values on the MSLT may adversely be affected by a spurious sleep latency on a single nap opportunity [28] possibly due to what might be perceived as stressful inter-nap activities [29], median values were also computed for each subject.

Measures of sleep architecture and diagnoses of primary sleep disorders were based upon data from MSLT and the second nocturnal polysomnography (to allow for sleep-lab habituation). Clinical outcomes of polysomnographic assessment and MSLT included obstructive sleep apnea, periodic limb movements, narcolepsy, insufficient sleep syndrome, primary/secondary insomnia, delayed sleep phase syndrome, bruxism, central sleep apnea, and upper airway resistance syndrome.

The polysomnographic outcome variables used in our analyses included: *total sleep time *(TST) (in min), *sleep efficiency *(% of time spent in bed asleep), *the percentage of TST spent in non-REM (NREM) and REM sleep, latency to sleep onset *(in min) to three consecutive epochs of sleep, and *REM latency*, defined as the time between the first epoch of any stage of sleep and the first epoch of REM-sleep. *Brief arousals *were scored following criteria set forth by the American Academy of Sleep Medicine, and the *number of arousals *expressed as a rate per hour of sleep adjusted for TST. *Periodic leg movements *both with and without accompanying arousals, were scored according to conventional criteria [30], and expressed as an index of the rate of leg movements per hour of sleep, and a separately derived index of those accompanied by an American Academy of Sleep Medicine -defined arousal [31]. We further recorded *alpha intrusion*, which was noted in review of 30-second segments.

Polysomnography data were scored by an Emory University registered polysomnology technologist and interpreted by an Emory University Department of Neurology American Board of Sleep Medicine certified physician [12].

### Assessment of subjective sleep quality and sleepiness

During the afternoon of their arrival at the hospital, subjects completed a self-administered questionnaire that explored themes and beliefs regarding sleep. The first two sleep specific questions, taken from the CDC Symptom Inventory [23], queried frequency and intensity of unrefreshing sleep and problems sleeping during the past month. A score of 0 reflected no difficulty with unrefreshing sleep or no problems sleeping and the maximum score of 16 indicated the problem had occurred all the time and was severe [see [23]]. The remaining 24 items of this questionnaire came from the Epworth Sleepiness Scale [32], which evaluates levels of excessive daytime sleepiness, and from the Toronto Sleep Assessment Questionnaire (SAQ^©^) [33], which measures self-reported sleep quality.

Subjects completed four questionnaires (the Nap Booklets) after each nap on day 1, which assessed latency to fall asleep during each nap. Subjects also completed two questionnaires (the Sleep Booklets) the morning after each overnight study, which evaluated 1) perceived sleep quality the night before on a visual analogue scale from 'Best possible sleep' (equals 0) to 'Worst possible sleep' (equals 140); 2) latency to fall asleep (in min); and 3) total sleep time (in min).

### Statistical analysis

Differences in categorical demographic data between CFS cases and non-fatigued controls were evaluated by Chi-Square or Fisher's exact test and continuous variables were compared by the *t*-test. Chi-Square test was also used for comparison CFS cases and non-fatigued controls in sleep study alterations. We used standard logistic regression analysis to regress CDC Symptom Inventory scores (unrefreshing sleep, problems sleeping) as well as Sleep Booklet scores (latency to fall asleep, total sleep time, sleep quality) and sleep medication use (yes/no) on case status (CFS/non-fatigued). Data from all participants was evaluated by logistic regression; in addition the subgroup of subjects with no alterations noted in sleep studies (normal sleep) were evaluated separately.

A two factor analysis of variance (ANOVA) using a general linear model was employed to measure the association between cases status and medication use (yes/no) with polysomnographic variables. Log transformed values of polysomnographic variables were used when necessary to satisfy the assumption of normally distributed outcomes. Mean values for each polysomnographic variable were adjusted for medication use by utilizing the least squares method.

Paired samples *t*-tests were used to compare 1) mean sleep latency, as measured by the MSLT, and mean sleep latency, as evaluated by the Nap Booklets and 2) latency to fall asleep and total sleep time as measured by nocturnal polysomnography with latency to fall asleep and total sleep time as measured by the Sleep Booklets. Comparisons were done separately for the group of subjects with CFS and for the non-fatigued controls. P-values for the paired *t*-tests were adjusted for multiple comparisons using both a Bonferroni correction and by computing a false discovery rate [34].

Sleep questionnaire data from the SAQ^© ^and the Epworth sleepiness scale were z-transformed for multivariate analyses. We used Principal Component Analysis (PCA) [35] with varimax rotation to evaluate which constellation of sleep symptoms represented the majority of the variance in sleep symptoms. Two-factor ANOVA was applied for comparison of factorial scores of sleep questionnaire items between CFS and non-fatigued groups, controlling for sleep medication use (yes/no). Comparison of factorial scores was done for all participants as well as for the subgroup of subjects with *normal *sleep studies.

Statistical significance for all tests was set at the 5% level. All statistics were computed using SPSS 12.0 (SPSS Inc, Chicago, IL).

## Results

Clinically significant apnea and narcolepsy (exclusionary for CFS) were diagnosed in 11 subjects based on overnight and daytime polysomnographic studies [12]. These subjects were not included in this analysis.

The remaining CFS and control subjects were demographically comparable. Thirty (85%) of the 35 with CFS and 36 (90%) of the 40 controls were women; 32 CFS (91%) and all controls were white; their mean ages were 50.3 (range 27 – 69) and 50.5 (range 32 – 65) years, respectively; and mean body mass index was 28.7 and 29.2, respectively. Medication use was more common in CFS subjects compared to non-fatigued controls; 20 CFS subjects (57%) compared to 5 control subjects (13%) took medications that alter sleep.

### Polysomnographic findings in CFS and non-fatigued

Detailed polysomnographic findings have been reported in detail [12]. In brief, previously undiagnosed sub-clinical sleep disorders occurred similarly in both CFS and non-fatigued controls (Table 1). Minimal obstructive sleep apnea and periodic limb movements were the most common alterations and occurred similarly among those with CFS and the controls. MSLT results were also comparable between the two groups. Finally, there were no statistically significant differences in standard polysomnographic measurements between those with CFS and non-fatigued controls on either night 1 or night 2. Since the first night served as an adaptation to the sleep laboratory, Table 2 summarizes only the night-2 data adjusted for medication use. In addition, each group appeared to experience similar periods of wakefulness during the study night as recorded in the % wakefulness during the sleep period.

**Table 1:** Sleep disorders in CFS and non-fatigued controls

***Sleep Disorders***†	**CFS (35)**	**NF (40)**	p*
*Obstructive Sleep Apnea (borderline)*	3 (8%)	3 (7%)	/
*Periodic Limb Movements*	7 (20%)	8 (20%)	NS
*Insufficient Sleep Syndrome*	1 (2%)	0	NS
*Delayed Sleep Phase Syndrome*	0	1 (2%)	NS

*MSLT Normal*	16 (45%)	16 (40%)	/
*MSLT Borderline*	13 (37%)	15 (37%)	NS
*MSLT Pathological*	6 (17%)	9 (22%)	NS

*Any Sleep Study Alteration*	26 (48%)	28 (52%)	NS

* Chi-square test.

NF = Non-fatigued; MSLT = Multiple Sleep Latency Test.

† No cases of Bruxism, Central Sleep Apnea or Upper Airway

Resistance Syndrome were identified.

**Table 2:** Sleep architecture in CFS and non-fatigued controls – Night 2 adjusted for medication use

	**CFS** **n = 35**	**NF** **n = 40**	p-value**
	Adjusted Mean*	Adjusted Mean*	

Total sleep time (min)	400.3	407.9	0.52
Sleep period time (min)	453.8	457.8	0.79
Latency to sleep onset (min)	21.3	17.1	0.47
REM latency (min)	98.4	106.8	0.40
Sleep efficiency (%)	88.3	90.2	0.32
Wake after onset (min)	53.8	44.0	0.69
Wake % Sleep Period	11.7	9.8	0.72
# Arousals	105.7	110.2	0.81
Arousal index	15.9	16.3	0.82
Stage 1 (% TST)	9.6	9.5	0.79
Stage 2 (% TST)	48.2	50.8	0.28
Stage 3/4 (% TST)	19.9	17.4	0.20
REM (% TST)	22.3	23.3	0.98
Alpha intrusion	0.29	0.49	0.11

*Mean values adjusted for medication use (yes/no).

** p-values generated using 2-factor analysis of variance

### Sleep symptoms in CFS and non-fatigued

Our analysis included two questionnaire items from the CDC Symptom Inventory that assess subjective sleep qualities over the preceding month, unrefreshing sleep and problems sleeping (getting to sleep, not sleeping through the night, or waking up on time), as well as one question from the Sleep Booklet, evaluation of sleep quality (best possible sleep to worst possible sleep) during the PSG. In a logistic regression analysis, we found an association of CFS with higher frequencies of symptoms of unrefreshing sleep and problems sleeping (p < .001 for each item) as well as worse ratings of sleep quality (p < .05); these associations remained after adjusting for use of medications that affect sleep.

Among subjects with *normal *objective sleep studies, those with CFS still reported significantly higher frequencies of unrefreshing sleep and problems sleeping than did non-fatigued controls (p < .001 for each item). In addition, CFS subjects with *normal *sleep studies also rated their quality of sleep during night #2 significantly worse than non-fatigued controls (p < .05).

### Perception versus polysomnographic assessment of sleep in CFS and non-fatigued

No significant differences between self-reported, as evaluated by the Nap Booklet, and the objective mean sleep latencies, recorded by the MSLT, were found for CFS subjects (Nap booklet score (± SE): 9.3 (± 0.9) minutes versus MSLT score : 7.2 (± 0.7) minutes, respectively; *t *(7) = 1.7. p = .13). In contrast, in non-fatigued controls, self-reported mean sleep latency was significantly longer than recorded mean sleep latency, MSLT score (± SE): 10.8 (± 1.5) min versus Nap booklet score :5.8 (± 0.6) min, respectively; *t*(16) = 2.9, p < .01).

Similarly, self-reported mean latency to fall asleep in non-fatigued controls, as reported in the Sleep Booklets, was significantly longer than mean latency to fall asleep, as measured by overnight polysomnography. These differences were found both on night #1 and night #2 in control subjects, but were more pronounced on night #1. The mean latency to fall asleep on night #1 was 18.9 (± 3.5) minutes as measured by PSG, versus mean latency to fall asleep night described in the Sleep booklet 31.8 (± 5.2) minutes (*t*(38) = 3.05, p < .005). The mean latency to fall asleep on night #2 was 16.6 (± 3.5) minutes as measured by PSG, versus latency to fall asleep night described in the Sleep booklet of 23.7 (± 4.1) minutes (*t*(38) = 2.4, p < .02). In contrast, no significant differences between subjective and objective latency to fall asleep during overnight polysomnography, were found in CFS subjects on either night #1 or night #2. These results remained even after excluding those subjects taking medications that affect sleep. There was no significant difference in total sleep time, as estimated by the Sleep Booklets, and total sleep time, as measured by overnight polysomnography, in either non-fatigued controls or CFS subjects.

Using the conservative Bonferroni correction for multiple comparisons at the α = .05 level, only the difference in night #1 sleep latencies in control subjects would remain significant. However, using the method of Benjamini and Hochberg and controlling the false discovery rate to < 10%, then all 3 of the p-values reported above are still significant [33] Together, these data suggest that altered perception of the latency to sleep onset is common in non-fatigued controls, but not in CFS patients.

Considering all items assessed by the SAQ^© ^and Epworth sleepiness scales, Principal Component Analysis (PCA) revealed six factors that accounted for the majority of variability in responses on the sleep questionnaire items. Table 3 shows the individual items comprising the six factors after a Varimax rotation with Kaiser normalization, the mean factor scores, and p-values for the differences between CFS and non-fatigued controls. A higher mean value for a factorial score represents more endorsement of the sleep questionnaire items comprising the factor (i.e. more sleep complaints). Factor score names were assigned to groups of questions comprising the different groupings based on the domains covered by the individual questions even though the questions were not designed with specific disorders or disturbances in mind. CFS cases had significantly higher scores in the *Insomnia *and *Physical/Somatic *factors compared to non-fatigued controls. CFS cases also had notably higher scored on the *Sleepiness *factor, although the difference was not statistically significant.

**Table 3:** Mean (SD) factorial scores and p-values for sleep questionnaire items in CFS and non-fatigued subjects

**Factor pattern on sleep questionnaire items**	**CFS (n = 35)**	**NF (n = 40)**	P *
	**Mean factor scores (SD)**^†^	**Mean factor scores (SD)**	

*F1 Insomnia*	0.54 (0.8)	-0.48 (0.9)	.000
"How often trouble sleeping", "Waking up before you wanted to", "Sleeping for less than 5 hours", "Difficulty falling asleep", "Repeated awakenings", "Waking up not feeling refreshed", "Restlessness during sleep"			

*F2 Sleepiness*	0.27 (1.2)	-0.24 (0.5)	.060
"Falling asleep while sitting and talking", "Falling asleep while doing something, such as driving or talking", "Falling asleep in a car while stopped in traffic", "Falling asleep while sitting and reading", "Falling asleep as a passenger in a car", "Falling asleep while sitting quietly after a lunch", "Falling asleep while sitting inactive in a public place", "Trouble staying awake"			

*F3 Physical/Somatic*	0.54 (1.0)	-0.48 (0.5)	.001
"Nightmares or waking up frightened or crying out loud", "Waking up with aches, pains, or stiffness", "Sleeping more than nine hours", "Taking medication for sleep"			

*F4 Apnea *"Interruptions to your breathing during sleep", "Falling asleep while lying down to rest in the afternoon"	0.09 (0.7)	-0.08 (1.2)	.865

*F5 Body Clock*	0.13 (1.2)	-0.11 (0.7)	.610
"Working shifts", "Irregular bed time and/or wake-up time during the work week or weekdays"			

*F6 Nasal Obstruction*	0.13 (1.1)	-0.12 (0.8)	.318
"Loud snoring"			

* 2-factor ANOVA, controlling for medication that influences sleep.

^† ^A higher factor score represents more agreement on the sleep questionnaire items comprising the factor (i.e. more sleep complains)

Differences in perception of sleep quality were even more pronounced between CFS cases and controls with *normal *objective sleep studies. CFS cases not only had significantly higher scores in the *Insomnia *(CFS: 0.51, non-fatigued: -0.56, p = 0.001) and *Physical/Somatic *(CFS: 0.41, non-fatigued: -0.42, p = 0.013) factors, but also in the *Sleepiness *factor (CFS: 0.39, non-fatigued: -0.27, p = .004).

## Discussion

The major finding of this study is the documentation of the extent and nature of sleep complaints experienced by CFS subjects compared to non-fatigued controls in the absence of differences in quantitative polysomnography and multiple sleep latency testing between the two groups. These findings are in agreement with previous clinic-based studies indicating that CFS patients perceive poor sleep in the absence of objective underlying sleep pathology [11,12,15,16]. However, the somewhat paradoxical observation that controls and not CFS subjects, overestimated the time to fall asleep, has not been previously reported and deserves further exploration. This finding suggests that CFS subjects may more closely monitor their sleep behaviour and that may contribute to their perceived sleep problems. It is also possible that persons with CFS are more accurate in their perceptions of their generally impaired sleep than people who do not have insomnia (but may sleep badly from time to time). This finding should be validated in further studies.

Even though identification of insomnia per se was not a goal of the study, it is interesting to note that CFS subjects in this study who were identified by the presence of a prolonged syndromic illness and its consequences also fulfilled a general definition for insomnia [9,18,36]. The symptom variables related to sleep (unrefreshing sleep and the 3 components of problems sleeping- getting to sleep, not sleeping through the night, or waking up on time) were identified by the patients themselves during the construction of the CFS symptom inventory [23]. Do these observations suggest that the CFS subjects have a problem with sleep efficacy, or that their descriptions of symptom association or our efforts to obtain information from them are inadequate? Are the CFS subjects identifying their impairments in terms of sleep based on the types of questions being asked with responses indicative of sleep problems not detected in the usual measures of sleep architecture?

These findings argue against the importance of readily identifiable sleep pathology contributing to the symptoms of CFS in the majority of CFS subjects. However, sleep disorders that may respond to clinical intervention should be evaluated in patients complaining of fatigue, and formal sleep studies are required in the evaluation of patients with suspected sleep disturbances. In clinical practice these disorders would have been considered as temporary exclusions of CFS and the patient re-evaluated after clinical re-evaluation [8]. New clinical interventions in CFS patients await further delineation of possible mechanisms required to explain these differences, but they will likely be based on pharmacological and/or behavioural modalities. However, such interventions need to be based on a better understanding of sleep physiology and the influences of chronic illness and exclusion of primary sleep disorders.

[0]Besides the theoretical issues addressed above, the present study is not without practical limitations. First, due to stringent selection criteria, our sample size was small, especially considering the number of variables examined. This circumstance limited the power to detect more subtle differences in responses to sleep questionnaire items between groups. Both CFS subjects and controls showed moderately impaired sleep quality (being in a research setting likely impaired sleep quality equally for both groups) and polysomnography is not an optimal measure of insomnia. Further studies with larger sample sizes are clearly warranted. Second, while sleep-altering medications were frequently used by both CFS subjects and controls, their use was more common among CFS subjects. Prescribed medications in CFS subjects may in turn have influenced CFS subjects' reports of sleep quality. Many published studies of sleep in persons with CFS do not consider medication use. Our attempt to statistically adjust for differences in use of medications that affect sleep as a binary measure (use/non-use) might be inadequate to completely control for the confounding effect of medication use. However, our small sample size precluded conducting a stratified analysis among cases and controls who did and did not use medications that alter sleep or whether they medications induced or inhibited sleep. Finally, the mean duration of illness among CFS cases in this population was 7.3 years [19]. Thus, findings in this study of prevalent CFS cases may not be applicable to those with a shorter duration of illness.

## Conclusion

These findings suggest that alterations in standard objective sleep parameters do not explain the etiology of symptoms of unrefreshing sleep and presence of sleep problems reported by persons with CFS who do not have readily diagnosable sleep disorders. Further studies examining the causes of apparent altered sleep-state perception may be helpful in understanding CFS.

## Competing interests

The author(s) declare that they have no competing interests.

## Authors' contributions

WCR and CH were principal investigators of the study. MM, CH, MJD, and WCR wrote the manuscript. All authors contributed to the manuscript. WCR, CH, ERU, LSY, JFJ, and MJD designed the study protocol and supervised data collection during the study. MJD designed sleep protocols, trained sleep technicians, and supervised sleep studies. MJD, CH, WCR, JFJ supervised the conduct of clinical studies and data collection. MM and MJD conducted the statistical analyses with assistance from BMG.

## Pre-publication history

The pre-publication history for this paper can be accessed here:


